# Cell line authentication: a commercial service provider perspective

**DOI:** 10.3389/fcell.2026.1843943

**Published:** 2026-07-09

**Authors:** Eleanor Ralston, Charlotte Haskayne

**Affiliations:** 1 Biofortuna Limited, Deeside, United Kingdom; 2 NorthGene™, Deeside, United Kingdom

**Keywords:** authentication, cross-contamination, misidentification, mixture analysis, primary cell lines, short tandem repeat (STR) profiling

## Abstract

Cell line misidentification and cross contamination continue to undermine research quality despite improved laboratory practices. Short Tandem Repeat (STR) profiling remains the primary method for human cell line authentication and is increasingly required by journals, funders, and regulatory bodies. Analysis of authentication data generated at NorthGene™ in 2024–2025 demonstrates that misidentification and contamination remain prevalent, with 4.7% of lines misidentified and 1.8% contaminated in 2024, and 2.4% misidentified with 1.6% contaminated in 2025. Primary cell lines present additional challenges, as none submitted during this period met ASN 0002 standards for full traceability, posing risks to reproducibility and downstream biological interpretation. Mixture analysis proved essential for resolving inconclusive STR results and identifying specific contaminants, although progress is hindered when reference STR profiles are not publicly available. While STR profiling is highly discriminatory for human cells, complementary methods—such as species specific PCR and CO1 barcoding—may be required to detect interspecies contamination or authenticate non human lines. Overall, these findings highlight the ongoing need for rigorous authentication workflows and complete traceability to ensure scientific integrity and prevent invalid research outcomes.

## 1 Introduction

Cell lines that have been mislabelled or contaminated with another cell line are important to identify. Misidentified cell lines lead to invalid research results, wasted resources, and potentially incorrect conclusions (Weiskirchen, 2026). DNA profiling via Single Tandem Repeat (STR) analysis ensures the cell line matches its original identity. DNA authentication can detect contamination early, preventing compromised experiments. In addition, testing can also confirm the type(s) of contaminating cell line assisting with laboratory investigations and preventing reoccurrence. Cost-benefit modelling has estimated that the expected Return on Investment over 5 years of performing STR authentication would be over 3,200% (Weiskirchen, 2026).

Scientific reproducibility depends on using the correct biological materials. Authentication provides confidence that published results are based on the intended cell line and many journals, funding agencies, and regulatory bodies now require proof of cell line authentication to maintain research integrity (Weiskirchen et al., 2025).

Here we review data generated from cell line authentication cases tested in 2024 and 2025 to identify current trends and how this highlights concerns which might not be detected by smaller scale studies or research conducted at a singular organisation. Some larger scale studies and reviews of authentication do exist (Fusenig et al., 2017; Freedman et al., 2015; Huang et al., 2017), alongside broader reviews of other published studies (Korch and Varella-Garcia, 2018), however there is still evidence that studies are being conducted without authentication prior to submission of manuscripts (Horbach and Halffman, 2017), with the International Journal of Cancer counting 1,400 manuscripts which were not re-submitted past the authentication requirements in 2011–2013 and when re-examined in 2019–2022 there were still 216 misidentified cell lines present within the submitted manuscripts. (Fusenig et al., 2017; Souren et al., 2022). This is at least indicative of improvement, although there is still a way to go before the issue can be considered truly addressed. A more concerning issue is that when misidentification has been detected in the data for published manuscripts, there have been a limited number of retractions, with the more frequent response being to acknowledge the information in a new publication (Korch and Varella-Garcia, 2018). However this does not prevent the original publication from being cited, as the new publication is not always clearly linked to the original.

## 2 The relevance of cell line authentication by STR profiling today

Cross-contamination of cell cultures was identified over 50 years ago (Gartler, 1968). However, over the years, laboratory practices have improved and scientists are now more aware of the risks to their cell lines and experiments. This should have significantly reduced the likelihood of cross contaminated cells from being used both in research and commercially.

In 2024 and 2025 one thousand, eight hundred and ninety-three samples were sent to NorthGene™ for cell line STR profiling. One thousand, three hundred and twenty-eight of these samples were immortalised human cell lines sent for authentication. The remainder were predominately samples where full authentication was not requested, only an STR profile, with a small number of primary cell lines being sent for authentication. In 2024, in 4.7% of the immortalised human cell lines we determined that misidentification had taken place. In addition to this, 3.6% produced an inconclusive result, where the percentage match using the Tanabe algorithm was between 59% and 80%. When reviewed, 50% of these inconclusive matches were confirmed to be due to contamination from another cell line that was present on Cellosaurus (Expasy, 2012). In 2025, 2.4% were classed as misidentified and a further 2.4% were inconclusive of which 66.7% were confirmed to contain contamination. These misidentified and contaminated cell lines were not present on the ICLAC Register of Misidentified Cell Lines (ICLAC, 2010), making it likely contamination happened within the laboratory culturing the cells and was not historical mislabelling or contamination. This confirms that the contamination of cell lines is still a very real issue today.

The numbers above contain an unknown but inherent bias as they come from organisations who routinely send the cells for STR-based authentication. A significant number of laboratories still do not undertake this practice (Freedman et al., 2015; Korch and Varella-Garcia, 2018) and are more likely to only check for microbial contamination (Freedman et al., 2015). Whilst we imagine that good cell-line practice is reflected in our numbers an underlying reason might be that cell lines are sent for authentication because they are suspected of being contaminated. Furthermore, the majority of cell line samples tested at our facility are sent from western Europe. Previous studies have detected a regional factor in the number of misidentified cell lines which this review is unable to account for (Fusenig et al., 2017; Huang et al., 2017; Korch and Varella-Garcia, 2018).

Several commercially available cell lines are known to be misidentified. The origin of contamination can date back as early as the 1960s, for example, the cell line WISH (Gartler and Westfall, 1967), which is still used today. However, even though reputable suppliers are clear that this cell line is now considered to be derived from HeLa, studies do still exist using these cells for the purpose of studying amniotic function, under the assumption that WISH is a representative human amnion-derived cell line (Pavan et al., 2003) and this is not a unique situation (Souren et al., 2022; Buehring et al., 2004; Kniss and Summerfield, 2014; Rojas et al., 2008; Korch et al., 2012). Where cell lines are from internal stocks or from another laboratory, there is a lesser chance of documentation confirming a historical contamination event and also of any quality checks performed on the cells prior to banking.

## 3 The importance of authenticating primary cell lines

For true traceability samples should be authenticated from primary culture. There is increased chance at this stage, firstly for a simple sample mix-up which may have occurred at the hospital, where the biopsy sample used is not associated with the donor who provided consent, and secondly, for an immortalised cell line to contaminate the primary cell line before any initial profiling has been performed (MacLeod et al., 1999). It is therefore recommended in ASN-0002 (Korch et al., 2021), which is a comprehensive standard widely accepted by international cell banks, to perform a threefold authentication; between a live sample from the donor, the biopsy sample and the cell line sample to confirm that no misidentification or cross contamination happened at any stage. However, this is often not the case. None of the requested primary cell lines received for testing in 2024 and 2025 were truly authenticated by that standard. The majority of cases received (28 out of 32) were for STR profile of the cell line only with no comparator sample. The remainder were match probability tested against samples from the original biopsy. This at least allows clear identification of the source tissue type, although not the donor. Of these, the majority were misidentified, however the subset is so small that this cannot be considered significant. The low numbers of primary cells tested is the issue of greater concern.

If the primary cell lines are not authenticated, this could invalidate any future studies which are dependent upon certain features of the cell type and the original tissue. If these experiments are used to assess the impact of an activity or pathway for a specific population it is important to prove that the sample came from a person within the population. False results could have a significant impact causing delays and resulting in rising costs (Weiskirchen, 2026; Freedman et al., 2015) and lead to a loss of trust from stakeholders.

The testing of primary cell lines are also becoming increasingly important. As time marches on and passage number increases, some characteristics of the cells have been known to change (Hughes et al., 2007). Many established cell lines do not have a documented passage number from the original primary cells. Therefore, it is impossible to determine the specific impact on the relevant characteristic and how this would differ from *in vivo* cells, making *in vitro* studies even more variable than is currently expected. This problem could be solved either by primary cell lines or with clearly documented cell lines with a limited number of passages. In addition, some cell types are not commonly available as immortalised cell lines therefore primary cell lines are needed in order to conduct investigations. In both cases, if the primary cell line or limited passage cell line includes a full traceable profile, where the cell line can be traced back to the original donor and sample type with each passage recorded, and where the original STR profile alongside any subsequent variations could be easily located, then the data would be more verifiable than within a large number of existing papers who have used cell lines immortalised prior to the introduction of authentication. It is not impossible that some frequently used cell lines were also contaminated or misidentified at the very beginning, which will now never be detected unless the contaminating profile is also available.

## 4 Why we recommend mixture analysis

In 2024 to 2025, 42 cell lines which were received for authentication at this facility generated an inconclusive result. Of these, 23 were confirmed to be contaminated with another human cell line which had an STR profile present on Cellosaurus using the Masters (vs. reference) algorithm, as recommended in Appendices E and F of ASN-0002 when searching for component cell lines in mixtures. Although some laboratories may simply discard potentially contaminated cell lines, it is beneficial to determine the contaminant particularly if the initial stock came from another laboratory or was a historical stock of their own. This allows for the contamination event to be narrowed down and could save other stocks of the cells which could then be used to conduct the experiment again once the source of contamination has been eliminated.

Mixture analysis is offered at this facility as a reflex test for intra-species contamination. It would be unlikely to pick up any non-human contamination as the primer set is designed for human STR sequences. Mixture analysis is not typically offered for samples which have been confirmed as misidentified, however occasionally our facility does advise customers to conduct mixture analysis even on samples which have been determined to be related to the expected cell line. An example is presented in Table 1, and in Supplementary Table 1.

**TABLE 1:** Table of allelic data, comparing sample D-1084796 against the expected STR profile on the Cellosaurus database using the Tanabe algorithm. The matching percentage was 83% which for 16 markers due to the failed marker was acceptable as ‘Related’. Mis-matched markers are flagged in red.

STR locus	CHP212 (test sample)	CHP-212 (RRID:CVCL_1125) (comparison profile)
D5S818	10, 11 , 12	10, 12
D13S317	8, 12 , 13	8, 13
D7S820	10 , 11	11
D16S539	12 , 13	13
vWA	15, 16 , 18	15, 18
TH01	6	6
TPOX	8, 11	8, 11
CSF1PO	12	12
AMEL	X, Y	X, Y
D3S1358	15, 17	15, 17
D21S11	27, 29, 32.2	27, 29
D18S51	14, 16	14, 16
Penta E	5, 11 , 15	5, 15
Penta D	9, 10, 13	9, 10
D8S1179	10 , 11, 13	11, 13
FGA	20	20
D19S433	FAIL	12, 15
D2S1338	22, 23 , 24 , 25	22, 25

**FIGURE 1:**
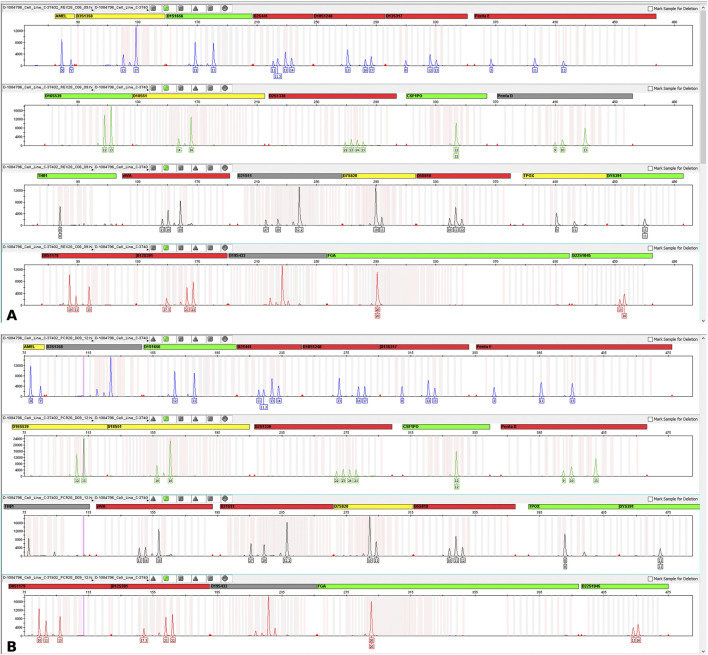
Electropherogram data from sample D-1084796 tested using PowerPlex ® Fusion kit, including retest. As D19S433 was outside of passing specifications for cell line samples for both runs, this was not included in the report. **(A)** Profile requiring re-check. **(B)** Repeat tested profile.

Although this was technically within the criteria for a ‘Related’ cell line, it was clear that it was likely the cell line was contaminated. The sample had already passed internal checks for contamination from within the run and from any staff members and had been repeat extracted to confirm that the profile was correct (Figure 1).

Microsatellite Instability (MSI) had been partially reviewed when locating the comparison profile on Cellosaurus. As there was a clear profile with no variation and the cell line had been deemed stable, this was not expected to be the cause of the additional peaks. However, MSI could not be completely ruled out as the cause of some of the additional peaks at this stage. A cover letter was issued with the report, recommending to the customer that they conduct mixture analysis on this sample.

The profile was reviewed. Any markers which had initially failed quality checks and any smaller peaks which were present but not called by the Genemapper® IDX v1.5 (RRID:SCR_021103) software were noted and incorporated into the mixture analysis alleles once the profile had been checked for bleed-through. These alleles were then uploaded into the CLASTR tool. The Masters (vs. reference) algorithm was selected, score filter set to 80% and minimum markers set to 13. The input file generated to upload to CLASTR, process figures and output file are provided in the Supplementary Material documents 2–4. Analysis confirmed 100% match contamination by NCI-H1299 (RRID:CVCL_0060) (Table 2).

**TABLE 2:** Profile comparison between expected cell line and the contaminating cell line. Using the Masters (vs. reference) algorithm, both cell lines had a matching percentage of 100%.

STR locus	CHP212 (test sample)	CHP-212 (RRID:CVCL_1125) (comparison profile)	NCI-H1299 (RRID:CVCL_0060) Contaminating cell line)
Amel	X, Y	X, Y	X
CSF1PO	12	12	12
D1S1656	12, 15	​	12, 15
D2S1338	22, 23, 24, 25	22, 25	23, 24
D2S441	11, 11.3, 13, 14	​	11, 13
D3S1358	15, 17	15, 17	17
D5S818	10, 11, 12	10, 12	11
D7S820	10, 11	11	10
D8S1179	10, 11, 13	11, 13	10, 13
D10S1248	13, 16, 17	​	13, 17
D12S391	17.3, 21, 22	​	21
D13S317	8, 12, 13	8, 13	12
D16S539	12, 13	13	12, 13
D18S51	14, 16	14, 16	16
D19S433	12, 14, 15	12, 15	14
D21S11	27, 29, 32.2	27, 29	32.2
D22S1045	15, 16	​	15, 16
FGA	20	20	20
Penta D	9, 10, 13	9, 10	13
Penta E	5, 11, 15	5, 15	11
TH01	6, 9.3	6	6, 9.3
TPOX	8, 11	8, 11	8
vWA	15, 16, 18	15, 18	16, 18

A negative match result would also prove useful. Contamination could potentially have come from the DNA of a scientist collecting the sample from the culture, highlighting issues with sample handling techniques, or from human cell lines with no or limited STR profiles present on Cellosaurus. We recommend that scientists working extensively with cell cultures should have their own STR profile on record.

## 5 The negative impact of proprietary information

The majority of commercial suppliers will provide a quality check certificate or a certificate of compliance clearly stating that the cell line is free from *mycoplasma* contamination and the cell line has been authenticated, often with the determined STR profile present. However, some laboratories maintain their own stocks of cells and do not make the STR profiles publicly available even if the cell line itself is present on Cellosaurus or other cell line collections (MacLeod and Drexler, 2006). Some suppliers will provide STR profiles upon request for organisations who have purchased that cell line, but others do not have an STR profile to provide and therefore there is no evidence that this cell line has not been contaminated or misidentified at any point previously. Although this is more common with academic institutions or hospitals, commercial providers are not exempt from this. In 2024, 5.5% of the samples received at our facility for cell line authentication did not have an STR profile publicly available, meaning authentication could not take place and instead only the STR profile was provided. In 2025, this increased to 7%.

Not adding STR profiles to a public dataset can cause problems when performing mixture analysis. It can be clear that a sample is contaminated but a significantly more complicated root cause investigation would be required to determine the true nature of the contamination.

Some laboratories do not make their authentication public but instead have internally maintained databases where samples are authenticated against their own previously grown cell lines. In these cases, it is recommended that the original cell line either follows the full primary cell line authentication path discussed previously if applicable, or is authenticated against a database. However, internal databases are a useful tool when the original supplier does not or cannot provide an STR profile. Traceability is not wholly complete as the sample cannot be proven to come from the source material but is at least present from the point of original upload.

In addition, although studies have been conducted, listing both misidentified cell lines and studies associated with misidentified cell lines, some of these studies are behind a paywall belonging to the journal, restricting public access (Nelson-Rees et al., 1981; Capes-D et al., 2010). Although these are readily accessible to most institutions, smaller companies may not wish to justify the expense, meaning checks on cell lines may not be performed prior to culturing. The ICLAC Register of Misidentified Cell Lines lists both cell lines where there is no authentic stock remaining and where authentic stock is believed to still exist, it is free to access. This register is regularly updated and is a useful tool for quickly checking whether a cell line is contaminated and prevents time wasted due to invalid results caused by historic cell line contamination.

Conversely, some cell lines, particularly those which have a high level of microsatellite instability, can have too much information provided. Each database handles variable STR profiles differently, for example, Cellosaurus lists all alleles with the sources who provided this data, where DSMZ CellDive (Dirks et al., 2010) lists each provided profile separately. It can be difficult to determine the source profile to ensure the current cell lines are authenticated against this. Most service providers instead put protocols in place to determine which profile to use to authenticate.

## 6 Determining the necessary level of authentication

STR profiling has been recommended in ASN-0002 to be the most suitable for human cell line authentication, as it is both cost-effective and can identify down to the individual, however there are still potential sources of contamination present which it cannot detect. We will ignore the possibility of microbial contamination for this discussion. Non-human cell lines do not have a standardised method for authentication, therefore it is currently left to the organisation to select the appropriate method (Alm et al., 2016).

The main drawbacks of STR profiling are that it cannot detect interspecies contamination, it cannot detect both species within a single test where a hybrid cell line is undergoing authentication and unless there is a traceable link to the original biopsy to where the user can be absolutely certain of source tissue it is not possible to determine tissue type. There are also a limited number of species which have both commercially available kits and a public database to perform authentication against. Some methods of authentication are challenging due to the need for specific equipment, such as fluorescence *in situ* hybridization but this can be used for detecting both intra- and inter species characteristics; isoenzyme analysis can be used for interspecies detection, however is not highly discriminatory, may not detect low levels of a contaminating species (Nims, 2018) and lacks commercially available reagents (Nims et al., 2018). Karyotypic analysis can detect both inter- and intra-species contamination, such as that in xenographs, however is time consuming and requires a higher level of expertise than that required for other technologies (Jacobsen et al., 2006; Korch et al., 2021).

Interspecies contamination can be checked by multiplex PCR where STR primers are not readily available for that species, however this is limited to the species that the primers have been designed to detect and cannot be used to identify individuals (Cooper et al., 2007). CO1 barcoding can be used to identify any species present on the Barcode of Life Database (Alm et al., 2016) or other similar databases, however cannot identify individuals and, unless performed using massively parallel sequencing, is only likely to detect the most prevalent species making it unhelpful for the detection of interspecies contamination (Cooper et al., 2007). During internal verification of CO1 barcoding in our laboratory only insect cell line contamination which has a less degenerate primer set was able to be distinguished from the main mammalian cell line. In addition, hybrids of sufficiently distantly related species often gradually lose one species of mtDNA with each passage, keeping only the mtDNA of the parent cell whose nuclear chromosomes were more stable (Attardi and Attardi, 1972; De Francesco et al., 1980). This would result in incomplete species detection for any test methods relying on mtDNA sequence for identification.

The requirements for cell line authentication should therefore depend upon the laboratory culturing the cells. Cell line authentication by STR profiling is recommended for human cell lines, however this may require supplementing with species-specific detection if the laboratory is also growing animal cell lines. Where the cell line in question does not have STR profiles determined, the most relevant species detection method should be selected.

## 7 Conclusion

Cell line authentication is an invaluable tool to ensure that current and future studies are not based on invalid data. Cell lines which have been truly validated from their primary cultures and provide complete traceability should be the aim of all users of cell lines, be they academic or commercial. Cell lines tested at NorthGene™ in the previous two calendar years confirm that contamination and misidentification is still a genuine problem even for institutions who do send their samples for authentication and this is likely significantly more prevalent for those who do not. Successful planning for a study should confirm that cell lines are suitable and where the supplier does not provide evidence of authentication it should be the responsibility of the institution to authenticate prior to culturing to prevent invalid study outcomes and to prove the integrity of their own scientific results.

## Data Availability

The original contributions presented in the study are included in the article/Supplementary Material, further inquiries can be directed to the corresponding author.

## References

[B1] Almeida J. L. Cole K. D. Plant A. L. (2016). Standards for cell line authentication and beyond. PLOS Biol. 14, e1002476. 10.1371/journal.pbio.1002476 27300367 PMC4907466

[B2] Attardi B. Attardi G. (1972). Fate of mitochondrial DNA in human-mouse somatic cell hybrids (density gradient centrifugation-ethidium bromide-karyotype). Proc. Natl. Acad. Sci. U. S. A. 69 (1), 129–133. 10.1073/pnas.69.1.129 4500544 PMC427560

[B3] Buehring G. C. Eby E. A. Eby M. J. (2004). Cell line cross-contamination: how aware are Mammalian cell culturists of the problem and how to monitor it? *in vitro* cell. Dev. Biol.-Animal 40, 211–215. 10.1290/1543-706X(2004)40<211:CLCHAA>2.0.CO;2 15638703

[B4] Capes-Davis A. Theodosopoulos G. Atkin I. Drexler H. G. Kohara A. MacLeod R. A. F. (2010). Check your cultures! A list of cross-contaminated or misidentified cell lines. Int. J. Cancer. 127, 1–8. 10.1002/ijc.25242 20143388

[B5] Cooper J. K. Sykes G. King S. Cottrill K. Ivanova N. V. Hanner R. (2007). Species identification in cell culture: a two-pronged molecular approach. Vitro Cell. Dev. Biol. Anim. 43 (10), 344–351. 10.1007/s11626-007-9060-2 17934781

[B6] De Francesco L. Attardi G. Croce C. M. (1980). Uniparental propagation of mitochondrial DNA in mouse-human cell hybrids. Proc. Natl. Acad. Sci. U. S. A. 77 (7), 4079–4083. 10.1073/pnas.77.7.4079 6254011 PMC349773

[B7] Dirks W. G. MacLeod R. A. Nakamura Y. Kohara A. Reid Y. Milch H. (2010). Cell line cross-contamination initiative: an interactive reference database of STR profiles covering common cancer cell lines, Int. Journal Cancer 126 (1), 303–304. 10.1002/ijc.24999 19859913

[B8] Expasy (2012). Cellosaurus - the reference resource on cell lines. Available online at: https://www.cellosaurus.org/.

[B9] Freedman L. P. Gibson M. C. Wisman R. Ethier S. P. Soule H. R. Reid Y. A. (2015). The culture of cell culture practices and authentication—results from a 2015 survey. BioTechniques 59 (4), 189–192. 10.2144/000114344 26458546

[B10] Fusenig N. E. Capes-Davis A. Bianchini F. Sundell S. Lichter P. (2017). The need for a worldwide consensus for cell line authentication: experience implementing a mandatory requirement at the international journal of cancer. PLoS Biol. 15 (4), e2001438. 10.1371/journal.pbio.2001438 28414712 PMC5393552

[B11] Gartler S. (1967). “Genetic markers as tracers in cell culture,” in National Cancer Institute Monograph 26: Second Decennial Review Conference on Cell Tissue and Organ Culture. Editor Westfall B. B. (Bethesda, MD: US. Department of Health Education and Welfare Public Health Service), 167–195. 4864103

[B12] Gartler S. (1968). Apparent HeLa cell contamination of human heteroploid cell lines. Nature 217, 750–751. 10.1038/217750a0 5641128

[B13] Horbach SPJM Halffman W. (2017). The ghosts of HeLa: how cell line misidentification contaminates the scientific literature. PLoS One 12 (10), e0186281. 10.1371/journal.pone.0186281 29023500 PMC5638414

[B14] Huang Y. Liu Y. Zheng C. Shen C. (2017). Investigation of cross-contamination and misidentification of 278 widely used tumor cell lines. PLoS ONE 12 (1), e0170384. 10.1371/journal.pone.0170384 28107433 PMC5249119

[B15] Hughes P. Marshall D. Reid Y. Parkes H. Gelber C. (2007). The costs of using unauthenticated, over-passaged cell lines: how much more data do we need? BioTechniques 43 (5), 575–586. 10.2144/000112598 18072586

[B16] ICLAC (2010). Register of Misidentified Cell Lines. Available online at: http://iclac.org/databases/cross-contaminations/[Currentreleasev14 (Accessed December 5, 2025).

[B17] Jacobsen B. M. Harrell J. C. Jedlicka P. Borges V. F. Varella-Garcia M. Horwitz K. B. (2006). Spontaneous fusion with, and transformation of mouse stroma by, malignant human breast cancer epithelium. Cancer Research 66 (16), 8274–8279. 10.1158/0008-5472.CAN-06-1456 16912208

[B18] Kniss D. A. Summerfield T. L. (2014). Discovery of HeLa cell contamination in HES cells: call for cell line authentication in reproductive biology research. Reprod. Sci. 21 (8), 1015–1019. 10.1177/1933719114522518 24520087 PMC4126216

[B19] Korch C. Varella-Garcia M. (2018). Tackling the human cell line and tissue misidentification problem is needed for reproducible biomedical research. Adv. Mol. Pathology 1 (1), 209–228.e36. 10.1016/j.yamp.2018.07.003

[B20] Korch C. Spillman M. A. Jackson T. A. Jacobsen B. M. Murphy S. K. Lessey B. A. (2012). DNA profiling analysis of endometrial and ovarian cell lines reveals misidentification, redundancy and contamination. Gynecol. Oncol. 127, 241–248. 10.1016/j.ygyno.2012.06.017 22710073 PMC3432677

[B21] Korch C. T. Hall E. M. Dirks W. G. Sykes G. R. Capes-Davis A. Barrett T. (2021). Human Cell Line Authentication Standardization of Short Tandem Repeat (STR) Profiling. Manassas, VA, USA: ATCC Standards Development Organization ASN-0002-2021.

[B22] MacLeod R. A. Drexler H. G. (2006). Public repositories: users reluctant to give materials. Nature 439 (7079), 912. 10.1038/439912b 16495971

[B23] MacLeod R. A. F. Dirks W. G. Matsuo Y. Kaufmann M. Milch H. Drexler H. G. (1999). Widespread intraspecies cross-contamination of human tumor cell lines arising at source. Int. J. Cancer 83, 555–563. 10.1002/(SICI)1097-0215(19991112)83:4<555::AID-IJC19>3.0.CO;2-2 10508494

[B24] Nelson-Rees W. A. Daniels D. W. Flandermeyer R. R. (1981). Cross-contamination of cells in culture. Science 212, 446–452. 10.1126/science.6451928 6451928

[B25] Nims R. W. (2018). Historical and current methods for detecting interspecies and intraspecies cell mixtures and thereby assuring cell line purity. Adv. Mol. Pathology 1 (1), 253–264. 10.1016/j.yamp.2018.07.005

[B26] Nims R. W. Capes‐Davis A. Korch C. T. Reid Y. A. (2018). Authenticating hybrid cell lines. Cell. Cult. 151–169. 10.5772/intechopen.80669

[B27] Pavan B. Fiorini S. Ferretti M. E. Vesce F. Biondi C. (2003). WISH cells as a model for the “in vitro” study of amnion pathophysiology. Curr. Drug Targets - Immune, Endocr. and Metabolic Disord. 3 (1), 83–92. 10.2174/1568008033340324 12570726

[B28] Rojas A. Gonzalez I. Figueroa H. (2008). Cell line cross-contamination in biomedical research: a call to prevent unawareness. Acta Pharmacol. Sin. 29, 877–880. 10.1111/J.1745-7254.2008.00809.X 18565286

[B29] Souren N. Y. Fusenig N. E. Heck S. Dirks W. G. Capes-Davis A. Bianchini F. (2022). Cell line authentication: a necessity for reproducible biomedical research. EMBO J. 41, EMBJ2022111307. 10.15252/embj.2022111307 35758134 PMC9289526

[B30] Weiskirchen R. (2026). Genetic insights into the economic toll of cell line misidentification: a comprehensive review. Med. Sci. (Basel). 14 (1), 25. 10.3390/medsci14010025 41562915 PMC12821653

[B31] Weiskirchen R. Almeida J. Chaqour B. (2025). Cell line authentication and validation is a key requirement for journal of cell communication and signaling publications. J. Cell. Commun. Signal 19 (2), e70029. 10.1002/ccs3.70029 40547562 PMC12181071

