# Supplementary material for: Cell line authentication: a commercial service provider perspective
Source: Front Cell Dev Biol. 2026 Jul 9;14:1843943. doi: 10.3389/fcell.2026.1843943 (PMC13391863; doi:10.3389/fcell.2026.1843943)
Supplement: Supplementary file 3 [file Table1.docx]

Supplementary Table 1

Table of allelic data as present on the issued report, comparing sample D-1084796 against the expected STR profile on the Cellosaurus database. The matching percentage was 83% which for 16 markers due to the failed marker was acceptable as ‘Related’.

Although homozygous alleles are present twice visually on the report in keeping with human profile reports, these are only counted as a single allele during calculations.

| **STR Locus** | **CHP212 (Test Sample)** | **CHP-212 (CVCL_1125) (Comparison Profile)** | **Match vs**  **Mismatch** |
| --- | --- | --- | --- |
| D5S818 | 10 \| 11 \| 12 | 10 \| 12 | Mismatch |
| D13S317 | 8 \| 12 \| 13 | 8 \| 13 | Mismatch |
| D7S820 | 10 \| 11 | 11 \| 11 | Mismatch |
| D16S539 | 12 \| 13 | 13 \| 13 | Mismatch |
| vWA | 15 \| 16 \| 18 | 15 \| 18 | Mismatch |
| TH01 | 6 \| 6 | 6 \| 6 | Match |
| TPOX | 8 \| 11 | 8 \| 11 | Match |
| CSF1PO | 12 \| 12 | 12 \| 12 | Match |
| AMEL | X \| Y | X \| Y | Match |
| D3S1358 | 15 \| 17 | 15 \| 17 | Match |
| D21S11 | 27 \| 29 \| 32.2 | 27 \| 29 | Mismatch |
| D18S51 | 14 \| 16 | 14 \| 16 | Match |
| Penta E | 5 \| 11 \| 15 | 5 \| 15 | Mismatch |
| Penta D | 9 \| 10 \| 13 | 9 \| 10 | Mismatch |
| D8S1179 | 10 \| 11 \| 13 | 11 \| 13 | Mismatch |
| FGA | 20 \| 20 | 20 \| 20 | Match |
| D19S433 | FAIL | 12 \| 15 | Excluded |
| D2S1338 | 22 \| 23 \| 24 \| 25 | 22 \| 25 | Mismatch |
