# Supplementary figures and images for: Cell line authentication: a commercial service provider perspective
Source: Front Cell Dev Biol. 2026 Jul 9;14:1843943. doi: 10.3389/fcell.2026.1843943 (PMC13391863; doi:10.3389/fcell.2026.1843943)

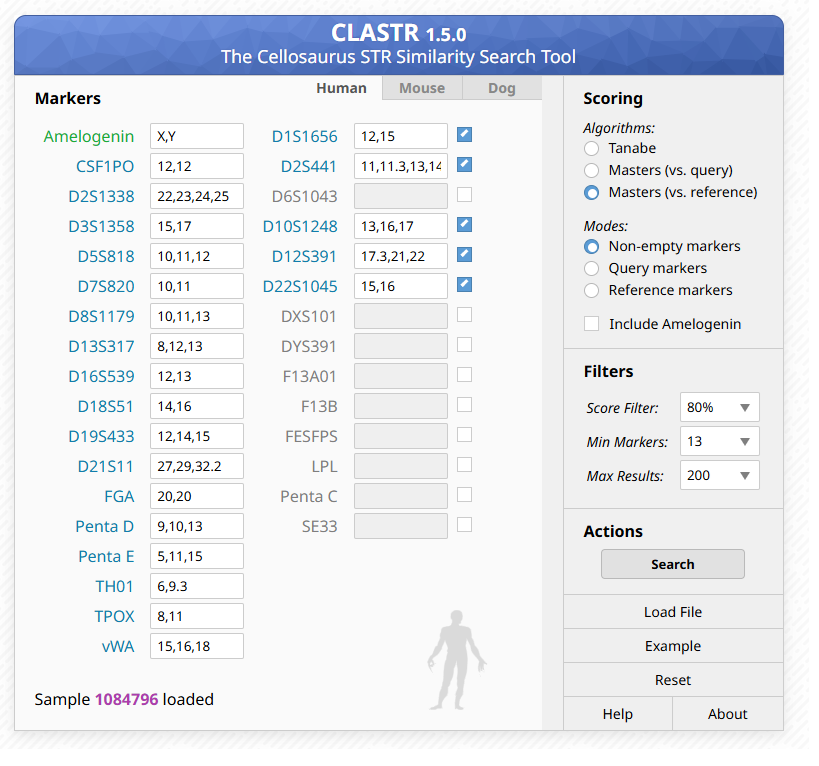

Supplement: Supplementary file 4 [file Image1.tif]
